# Supplementary material for: Bacterioplankton Communities in Dissolved Organic Carbon-Rich Amazonian Black Water
Source: Microbiol Spectr. 2023 May 18;11(3):e04793-22. doi: 10.1128/spectrum.04793-22 (PMC10269884; doi:10.1128/spectrum.04793-22)
Supplement: Supplemental file 1 — Supplemental material. Download spectrum.04793-22-s0001.pdf, PDF file, 2.7 MB [file spectrum.04793-22-s0001.pdf]

Supplementary material

Bacterioplankton communities in dissolved organic carbon-rich Amazonian black water.

Sylvain, François-Étienne; Bouslama, Sidki; Holland, Aleicia; Leroux, Nicolas; Mercier, Pierre-Luc; Val, Adalberto Luis; Derome, Nicolas

Supplementary figures

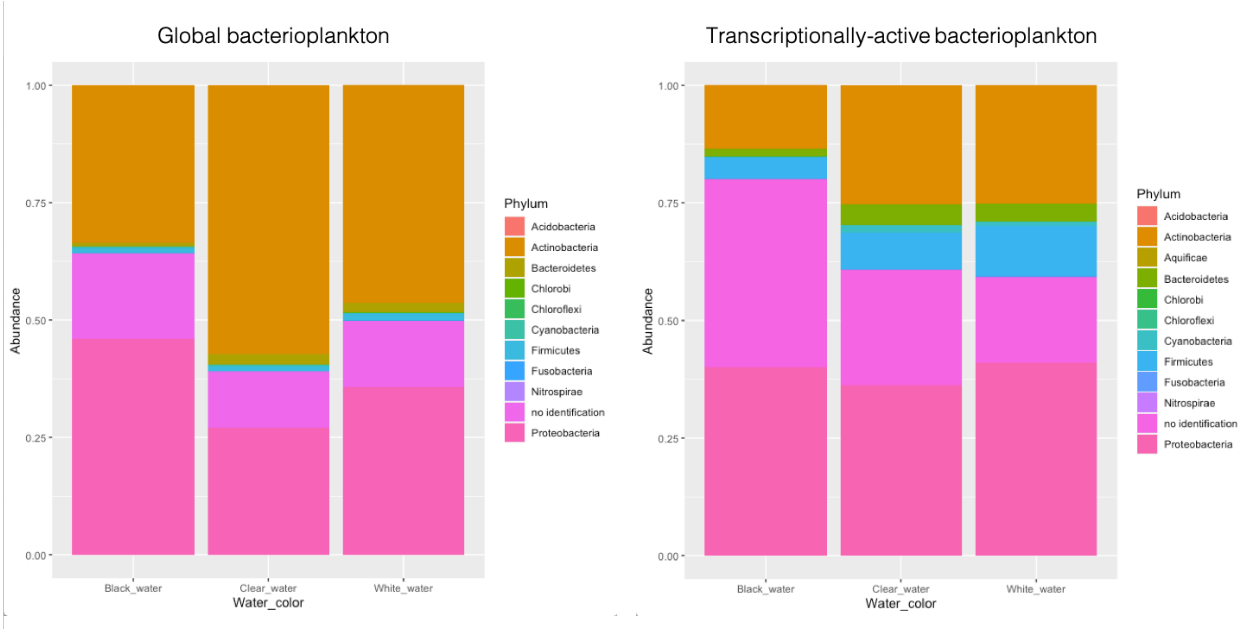

Suppl. Figure 1: Relative abundance of the most abundant phyla in global and transcriptionally-active bacterioplankton. Samples are grouped according to their water color.

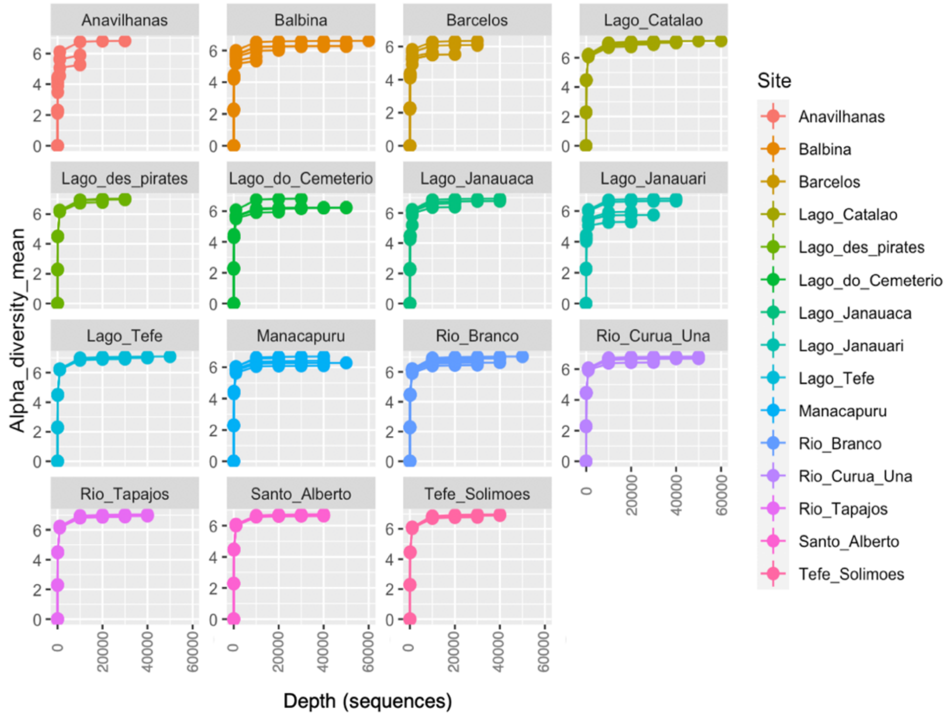

Suppl. Figure 2: Rarefaction plots of the samples for each sampling site, for the global bacterioplankton. The rarefaction analysis was based on the Shannon diversity for each sample group, according to the sequencing depth (number of sequences used).

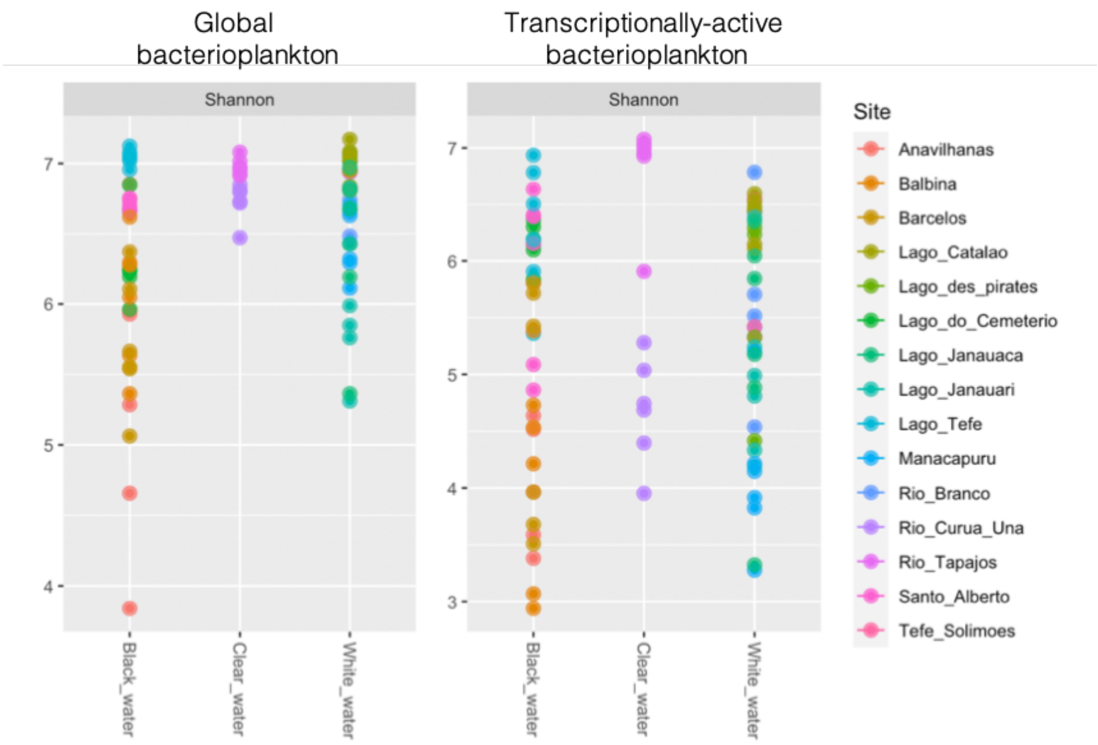

Suppl. Figure 3: Shannon diversity plots of the samples for the taxonomic structure of global and transcriptionally-active bacterioplankton. Samples are grouped according to their water color, and are colored according to their sampling site of origin.

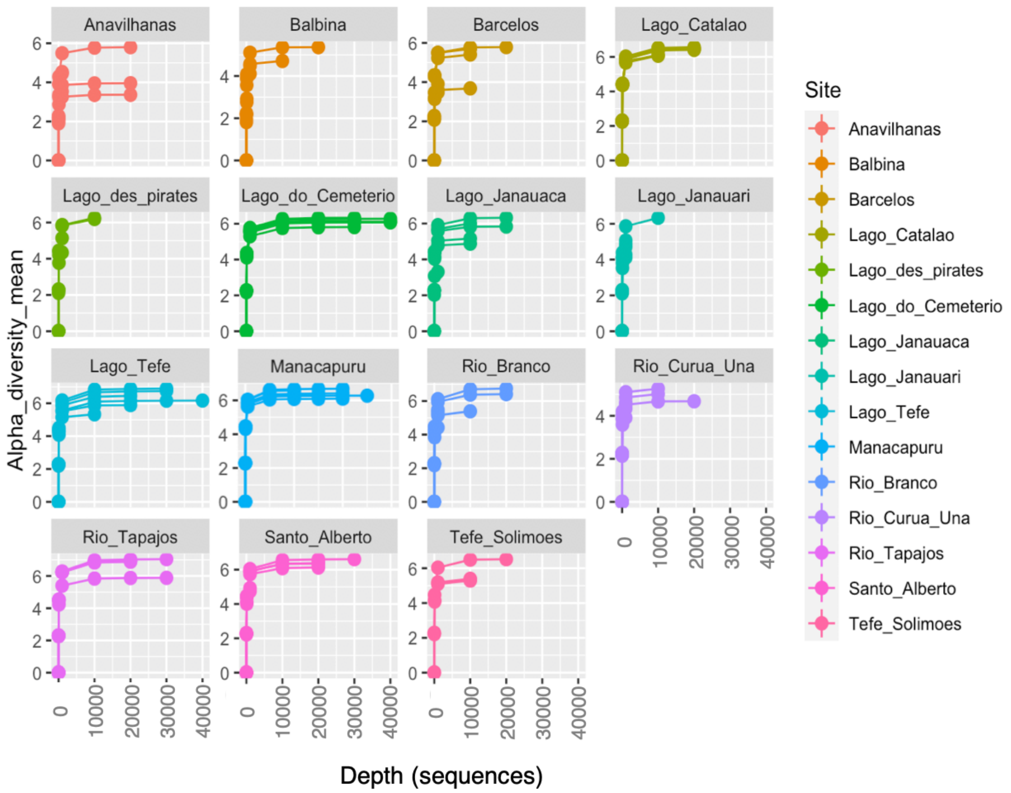

Suppl. Figure 4: Rarefaction plots of the samples for each sampling site, for transcriptionally-active bacterioplankton. The rarefaction analysis was based on the Shannon diversity for each sample group, according to the sequencing depth (number of sequences used)

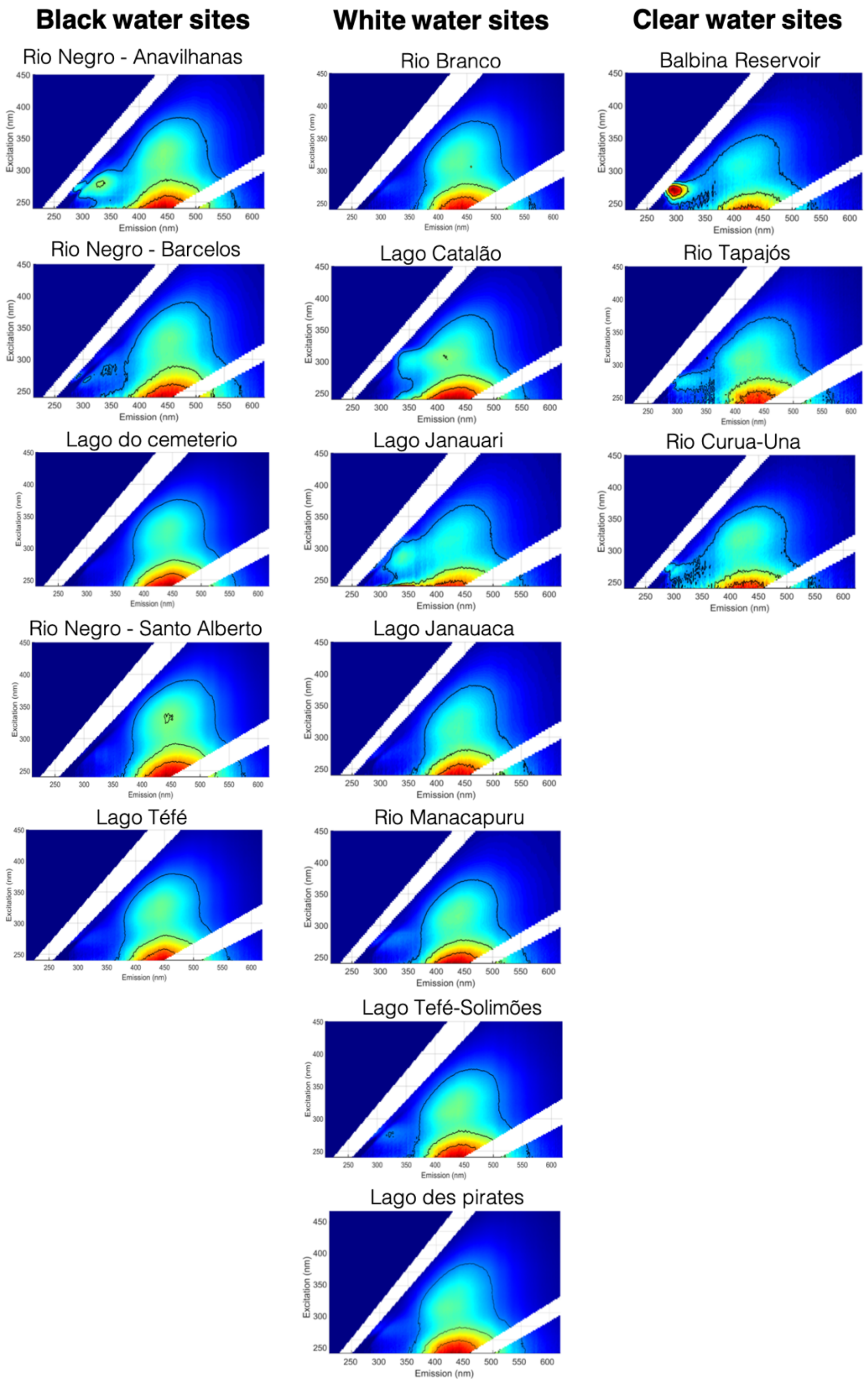

Suppl. Figure 5: Fluorescence Excitation Emission Scans (FEEMs) of the FDOM from all sites.

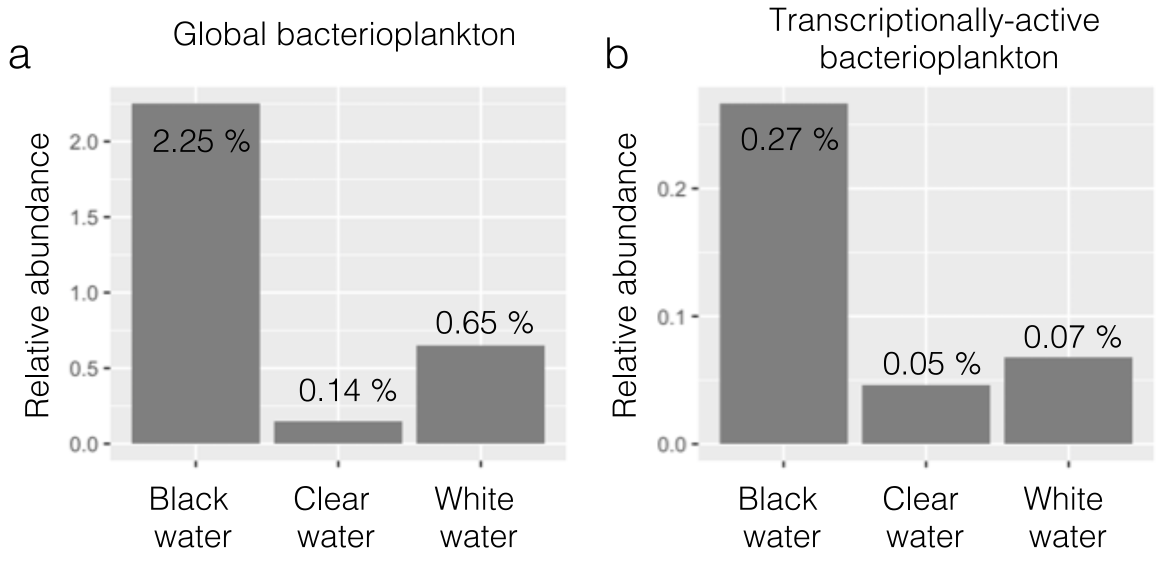

Suppl. Figure 6: Average relative abundance of *Polynucleobacter* ASVs in each water type for global bacterioplankton (a) and for transcriptionally-active bacterioplankton (b).

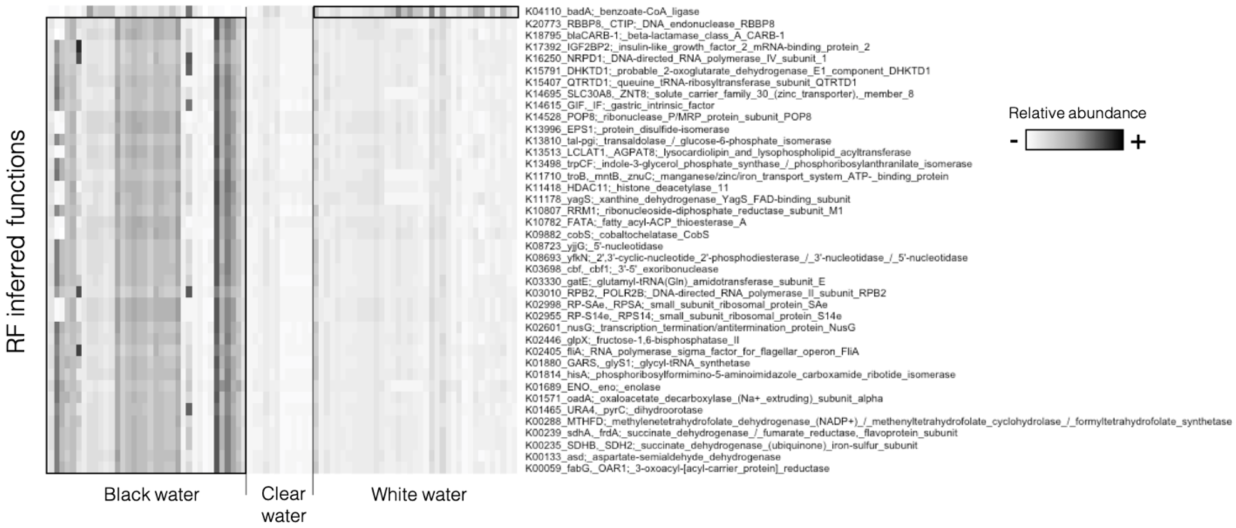

Suppl. Figure 7: Random-Forest (RF) machine-learning analysis identifies the 40 inferred functions showing the most important differentiation between black, clear and white water types. Heatmap columns represent samples and rows are different functions. Black boxes identify to which water type were associated to each inferred function.

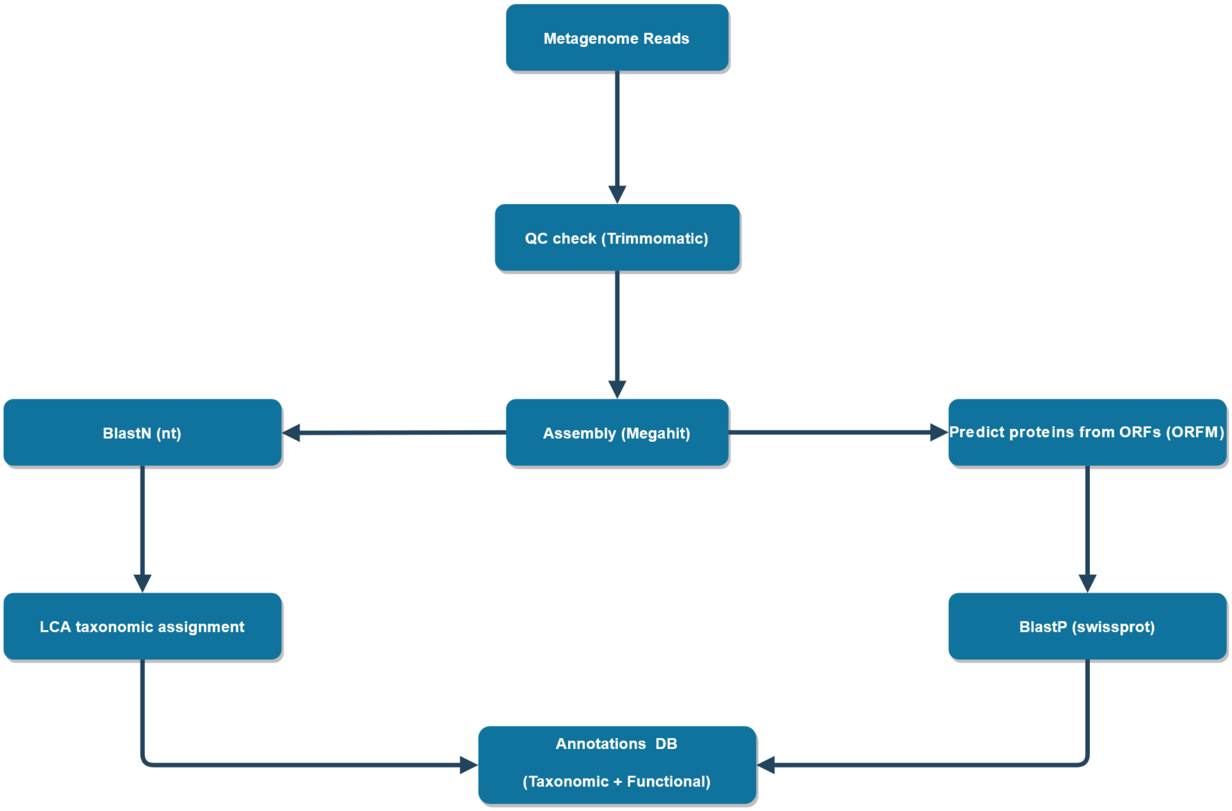

Suppl. Figure 8: Flowchart of the pipeline used to produce the functional reference database.

Supplementary Tables

Suppl. Table 1: Results from the Random Forest classification tests with error rates for each water type.

Classified as	Global bacterioplankton				Transcriptionally-active bacterioplankton				Inferred functions			
	Black water	Clear water	White water	Class error	Black water	Clear water	White water	Class error	Black water	Clear water	White water	Class error
Black water	36	0	0	0	25	0	0	0.31	30	0	6	0.17
Clear water	0	11	1	0.08	1	5	6	0.58	2	6	4	0.5
White water	0	0	37	0	5	0	32	0.14	7	1	29	0.22
	Out-of-bag error rate = 1.18 %				Out-of-bag error rate = 27.06 %				Out-of-bag error rate = 23.53 %			

Suppl. Table 2: Results of PERMANOVA tests conducted on bacterioplankton communities on the basis of the “Ecosystem” variable from Table 1.[1]

PERMANOVA : Lakes versus rivers				
**Water color**	**DNA or RNA**	**df res**	**F**	**p-value**
All	DNA	83	3.55	< 0.001
All	RNA	83	2.04	< 0.001
Black	DNA	34	5.02	< 0.001
Black	RNA	34	2.43	< 0.001
White	DNA	35	4.47	< 0.001
White	RNA	35	1.52	0.02

***1** : The groups used for the PERMANOVA tests consisted of “Lake” and “River” ecosystems. Here, “Water color” and “DNA or RNA” variables characterize the subset of samples that were used for the tests. “df res” means the number of degrees of freedom for residuals.

Suppl. Table 3: List of enzymes known to play a role in bacterial humic degradation processes from the literature.[1]

Step	KEGG ID	Enzyme name
Oxydation	K15733	E1.11.1.19; dye decolorizing peroxidase [EC:1.11.1.19]
Oxydation	K05909	E1.10.3.2; laccase [EC:1.10.3.2]
Oxydation	K17686	copA, ctpA, ATP7; P-type Cu+ transporter [EC:7.2.2.8]
Oxydation	K04564	SOD2; superoxide dismutase [EC:1.15.1.1]
Oxydation	K04565	SOD1; superoxide dismutase [EC:1.15.1.1]
Oxydation	K16627	SOD3; superoxide dismutase [EC:1.15.1.1]
Oxydation	K03782	katG; catalase-peroxidase [EC:1.11.1.21]
Oxydation	K23515	LPO; lignin peroxidase [EC:1.11.1.14]
Oxydation	K20205	mpn; manganese peroxidase [EC:1.11.1.13]
Oxydation	K20929	GLX; glyoxal/methylglyoxal oxidase [EC:1.2.3.15]
Oxydation	K17990	VCPO; vanadium chloroperoxidase [EC:1.11.1.10]
Oxydation	K21820	APO1; unspecific peroxygenase [EC:1.11.2.1]
Oxydation	K19813	gdh; glucose dehydrogenase [EC:1.1.5.9]
Oxydation	K23272	P2OX; pyranose oxidase [EC:1.1.3.10]
Oxydation	K19069	CDH; cellobiose dehydrogenase (acceptor) [EC:1.1.99.18]
Oxydation	K00432	gpx, btuE, bsaA; glutathione peroxidase [EC:1.11.1.9]
Funneling of monoaryls	K18383	ferB; feruloyl-CoA hydratase/lyase [EC:4.1.2.61]
Funneling of monoaryls	K12508	fcs; feruloyl-CoA synthase [EC:6.2.1.34]
Funneling of monoaryls	K05337	fer; ferredoxin
Funneling of monoaryls	K13310	desV, eryCI; dTDP-3-amino-3,4,6-trideoxy-alpha-D-glucose transaminase [EC:2.6.1.106]
Funneling of monoaryls	K21802	vdh; vanillin dehydrogenase [EC:1.2.1.67]
Funneling of diaryls	K15063	ligW; 5-carboxyvanillate decarboxylase
Funneling of diaryls	K15060	ligX; 5,5'-dehydrodivanillate O-demethylase
Funneling of diaryls	K15062	ligY; OH-DDVA meta-cleavage compound hydrolase
Funneling of diaryls	K15061	ligZ; OH-DDVA oxygenase
Funneling of diaryls	K00799	GST, gst; glutathione S-transferase [EC:2.5.1.18]
Funneling of diaryls	K22465	bzaA B; 5-hydroxybenzimidazole synthase [EC:4.1.99.23]
Funneling of diaryls	K21568	PLR; pinoresinol/lariciresinol reductase [EC:1.23.1.1 1.23.1.2 1.23.1.3 1.23.1.4]
Funneling of diaryls	K01971	ligD; bifunctional non-homologous end joining protein LigD [EC:6.5.1.1]
Funneling of diaryls	K01975	thpR; RNA 2',3'-cyclic 3'-phosphodiesterase [EC:3.1.4.58]
O-demethylation	K00297	metF, MTHFR; methylenetetrahydrofolate reductase (NADPH) [EC:1.5.1.20]
O-demethylation	K01938	fhs; formate--tetrahydrofolate ligase [EC:6.3.4.3]

O-demethylation	K15066	ligM; vanillate/3-O-methylgallate O-demethylase [EC:2.1.1.341]
O-demethylation	K15064	desA; syringate O-demethylase [EC:2.1.1.-]
O-demethylation	K23526	gcoA; aromatic O-demethylase, cytochrome P450 subunit [EC:1.14.14.-]
O-demethylation	K23527	gcoB; aromatic O-demethylase, reductase subunit [EC:1.6.2.-]
O-demethylation	K03862	vanA; vanillate monooxygenase [EC:1.14.13.82]
O-demethylation	K03863	vanB; vanillate monooxygenase ferredoxin subunit
O-demethylation	K14727	pcaL; 3-oxoadipate enol-lactonase / 4-carboxymuconolactone decarboxylase [EC:3.1.1.24 4.1.1.44]
Ring cleavage	K10221	ligI; 2-pyrone-4,6-dicarboxylate lactonase [EC:3.1.1.57]
Ring cleavage	K10218	ligK, galC; 4-hydroxy-4-methyl-2-oxoglutarate aldolase [EC:4.1.3.17]
Ring cleavage	K19338	nac; LysR family transcriptional regulator, nitrogen assimilation regulatory protein
Ring cleavage	K09788	prpF; 2-methylaconitate isomerase [EC:5.3.3.-]
Ring cleavage	K04099	desB, galA; gallate dioxygenase [EC:1.13.11.57]
Ring cleavage	K15065	desZ; 3-O-methylgallate 3,4-dioxygenase [EC:1.13.11.-]
Ring cleavage	K04100	ligA; protocatechuate 4,5-dioxygenase, alpha chain [EC:1.13.11.8]
Ring cleavage	K04101	ligB; protocatechuate 4,5-dioxygenase, beta chain [EC:1.13.11.8]
Ring cleavage	K10219	ligC; 2-hydroxy-4-carboxymuconate semialdehyde hemiacetal dehydrogenase [EC:1.1.1.312]
Ring cleavage	K10220	ligJ; 4-oxalmesaconate hydratase [EC:4.2.1.83]
Ring cleavage	K00448	pcaG; protocatechuate 3,4-dioxygenase, alpha subunit [EC:1.13.11.3]
Ring cleavage	K00449	pcaH; protocatechuate 3,4-dioxygenase, beta subunit [EC:1.13.11.3]

***1**: Retrieved from de Gonzalo *et al.* (2016), Kamimura *et al*. 2017, Santos *et al.* (2020).

Suppl. Table 4: Concentrations of free ions and nutrients.

Site #	Water color	Ions: mg L^{-1}					Nutrients: umol L^{-1}		
		Na^{+}	Mg^{+2}	K^{+}	Ca^{+2}	Cl^{-}	Nitrite	Nitrate	Silicate
1	Black	0.46	0.12	0.42	0.04	0.11	0.11	3.20	64.41
2	Black	0.25	0.09	0.33	0.49	1.16	0.10	2.87	92.32
3	Black	1.80	0.26	0.65	0.08	0.32	0.09	4.36	72.55
4	Black	0.23	0.05	0.14	0.37	0.64	0.01	0.47	76.82
5	Black	0.87	0.19	0.56	0.82	0.53	0.08	4.09	217.19
6	White	1.15	0.43	0.70	0.93	1.10	0.04	8.23	180.48
7	White	1.99	0.20	0.79	0.06	1.47	0.19	1.31	98.31
8	White	4.56	3.76	1.71	0.83	1.75	0.09	0.56	242.31
9	White	3.32	1.00	1.07	0.44	2.17	0.13	20.45	156.51
10	White	4.91	0.14	1.45	0.05	1.43	0.12	1.53	126.01
11	White	1.95	0.21	0.28	1.11	1.29	0.03	6.47	326.53
12	White	5.35	1.76	1.28	1.17	3.26	0.61	11.96	222.31
13	Clear	0.80	0.14	0.67	0.03	0.78	0.05	1.55	85.93
14	Clear	0.43	0.47	0.57	0.68	0.39	0.09	1.91	179.36
15	Clear	1.52	0.26	0.67	0.04	1.22	0.06	2.55	171.96

Suppl. Table 5: Primary productivity characterization and measure of several physicochemical parameters.[1]

Site #	Water color	Primary productivity: ug L^{-1}			Physicochemical parameters			
		Chl a	Phaeopig.	Chla/DOC	Temp. °C	Cond. uS	pH	% O_2
1	Black	0.35	2.43	0.03	31.60	13.10	3.71	92.12
2	Black	0.73	0.33	0.06	30.60	10.60	4.16	58.00
3	Black	0.05	0.38	0.00	30.70	13.20	4.24	53.20
4	Black	1.35	1.44	0.14	32.40	7.20	3.83	76.90
5	Black	1.82	1.73	0.26	30.00	10.60	4.98	61.50
6	White	6.21	2.89	1.03	31.00	22.00	6.25	88.70
7	White	4.62	17.31	0.65	32.90	22.40	4.38	60.00
8	White	7.14	6.60	0.79	32.90	174.80	5.70	44.00
9	White	1.35	1.88	0.24	29.30	88.00	6.75	82.60
10	White	2.78	10.54	0.35	32.60	24.30	5.31	72.80
11	White	4.41	3.20	0.77	30.30	19.70	6.05	68.60
12	White	9.05	4.69	1.40	31.90	127.60	7.15	31.90
13	Clear	0.83	0.78	0.17	33.20	16.80	5.05	103.20
14	Clear	2.15	1.03	0.81	30.00	14.10	6.36	80.20
15	Clear	1.25	2.38	0.28	31.20	19.00	6.00	79.10

***1:** "Chl a" means the concentration of chlorophyll a; "Phaeopig." means the concentration of phaeopigments; "Chla/DOC" is a ratio of the concentration of chlorophyll a divided by the concentration of DOC; "Temp. °C" means the temperature in ° Celsius; "Cond. uS" means the conductivity in microsiemens; "% O_2" means the percentage of saturation of dissolved oxygen.

Suppl. Table 6: Concentration of dissolved metals in ug/L.

Site #	Water color	Metals (ug/l)											
		Al	V	Cr	Mn	Fe	Co	Ni	Cu	Zn	As	Cd	Pb
1	Black	137.75	0.38	0.30	7.38	166.63	0.13	1.93	10.36	33.48	0.16	0.09	1.43
2	Black	150.00	0.10	0.05	5.90	160.00	0.10	0.15	0.30	11.00	0.05	0.02	0.27
3	Black	36.33	0.34	0.37	9.24	142.38	0.28	3.23	9.25	72.92	0.48	0.21	1.11
4	Black	87.00	0.30	0.05	4.60	100.00	0.10	0.33	1.90	9.00	0.08	0.13	0.30
5	Black	62.00	0.10	0.33	13.00	220.00	0.10	0.52	0.60	4.40	0.19	0.02	0.12
6	White	38.00	0.20	0.05	0.51	230.00	0.10	0.14	0.80	2.60	0.07	0.02	0.26
7	White	65.50	0.78	0.40	9.85	269.28	0.10	0.85	16.19	44.15	0.47	0.06	0.67
8	White	1.81	0.17	0.10	0.61	5.84	0.10	0.48	2.20	171.78	0.99	0.02	0.03
9	White	28.02	1.45	0.09	11.25	166.97	0.10	0.58	2.73	1.83	0.70	0.03	0.25
10	White	13.47	0.85	0.21	4.64	97.85	0.10	1.12	2.11	25.85	0.38	0.08	0.16
11	White	49.00	0.30	0.11	0.68	250.00	0.10	0.41	0.50	2.70	0.27	0.02	0.21
12	White	27.00	0.20	0.06	4.60	82.00	0.10	0.60	1.70	8.10	1.30	0.02	0.11
13	Clear	10.29	0.05	0.05	0.23	16.85	0.10	0.13	0.56	4.49	0.14	0.02	0.05
14	Clear	5.00	0.10	0.05	0.05	7.00	0.10	0.10	0.50	3.70	0.07	0.02	0.03
15	Clear	18.49	0.17	0.58	12.31	52.88	0.11	1.18	2.12	23.94	0.64	0.07	0.25

Supplementary results and discussion

Water residence time

Our results suggest that water residence time significantly influenced the taxonomic structure and transcriptional activity of the bacterioplankton communities, both in black and white water environments (results in Suppl. Table 1). Although water residence time was not specifically measured in this study, ecosystems were classified as lakes (longer residence time) and rivers (shorter residence time) and enabled us to perform PERMANOVA analyses based on the type of ecosystem that was studied (see Table 1). Water residence time moderates the extent and the time during which environmental pressures act on communities to create species sorting, or selection (Ben Maamar *et al.* 2015; Abbott *et al.* 2016; Niño-García *et al.* 2016; Jones *et al.* 2020). Longer residence times can potentially lead to feedback loops where bacterial communities engineer new conditions that select for different sets of taxa (Jones *et al.* 2020). For example, in longer residence times, anoxic conditions can be observed below the sediment-water interface if microbial decomposition exceeds the reaeration rate (Baker *et al.* 2000; Zarnetske *et al.* 2011). In such conditions, alternative terminal electron acceptor pathways are activated and can lead to a switch from net bacterial nitrification to denitrification processes affecting global water physicochemistry (Briggs *et al.*, 2013; Oldham *et al.* 2013; Kolbe *et al.* 2019). In the Amazonian River system, water residence time varies according to the intense seasonality experienced by these ecosystems: Between the rainy season (January to June) and the dry season (July to December) the Amazon water level can vary of several meters – a variation of 29 meters was recorded in June 2021 (Espinoza *et al.* 2022). The temporal variability modulates the connectivity between environments and thus the water residence time. Overall, the effects of this seasonality on watercourse residence time, connectivity, and chemical profile likely interferes with bacterioplankton communities and merits further investigation.

Pathways of humic compounds' degradation

The set of genes that was detected in Amazonian *Polynucleobacter*, *Methylobacterium* and *Acinetobacter* (Fig. 7) suggests that they possess the genomic potential to be involved in the degradation of humic acids or their by-products, via a derivative of the β-aryl ether degradation pathway for diaryl residues.

Polynucleobacter: The *Polynucleobacter* detected contained the glutathione S-transferases ligF/ligG (GST, K00799), performing one of the main reactions of this funneling pathway leading to the production of vanillate. The O-demethylation of vanillate is still unresolved based on the gene set detected, but could involve a demethylase similar to ligM (K15066), since we detected genes coding for enzymes associated with the metabolism of protocatechuate (PCA), the product of the ligM reaction, such as 3-oxoadipate enol-lactonase/4-carboxymuconolactone decarboxylase

(pcaL, K14727). The degradation of these substances appears to conclude in an extradiol 4,5-PCA ring meta cleavage as suggested by the presence of genes coding for enzymes ligI (a 2-pyrone-4,6-dicarboxylate lactonase, K10221) and ligK (a 4-hydroxy-4-methyl-2-oxoglutarate aldolase, K10218) associated with this pathway (de Gonzalo *et al*. 2016).

Methylobacterium: The set of genes found in *Methylobacterium* suggests that, like *Polynucleobacter*, this clade could degrade humic substances via a derivative of the β-aryl ether degradation pathway for diaryl residues, and concludes in an extradiol 4,5-PCA ring meta cleavage producing pyruvate and oxaloacetate.

Acinetobacter: In *Acinetobacter*, the O-demethylation of vanillate is achieved by a two-component monooxygenase composed of an oxygenase (VanA) and a reductase (VanB) (as detailed in Segura *et al*. 1999). Finally, in contrast to the extradiol 4,5-PCA ring meta cleavage of *Polynucleobacter* and *Methylobacterium*, we observed that *Acinetobacter* possessed the genes coding for the protocatechuate 3, 4-dioxygenase enzyme (pcaGH, K00448, K00449) associated to intradiol ring cleavage, as detailed in Vetting *et al*. (2000).

References

1. Abbott, B. W., and others. 2016. Using multi-tracer inference to move beyond single-catchment ecohydrology. Earth Sci. Rev. 160, 19–42. doi: 10.1016/j.earscirev.2016.06.014
2. Baker, M. A., Dahm, C. N., and Valett, H. M. 2000. Anoxia, anaerobic metabolism biogeochemistry of the stream water-ground water interface, p. 259–284. In J. B. Jones Jr. and P. J. Mulholland [eds.], Streams and Ground Waters. Academic Press.
3. Ben Maamar, S., and others. 2015. Groundwater isolation governs chemistry and microbial community structure along hydrologic flowpaths. Front. Microbiol. 6:1457. doi: 10.3389/fmicb.2015.01457
4. Briggs, M. A., Lautz, L. K., Hare, D. K., and González-Pinzón, R. 2013. Relating hyporheic fluxes, residence times, and redox-sensitive biogeochemical processes upstream of beaver dams. Freshw. Sci. 32, 622–641. doi: 10.1899/12-110.1
5. Callahan, B. J., McMurdie, P. J., Rosen, M. J., Han, A. W., Johnson, A. J. A., and Holmes, S. P. 2016. DADA2: High-resolution sample inference from Illumina amplicon data. Nature Meth. 13(7):581.
6. de Gonzalo, G., Colpa, D. I., Habib, M. H. M., and Fraaije, M.W. 2016. Bacterial enzymes involved in lignin degradation. J. Biotechnol. 236:110-9.
7. Espinoza, J. C., Marengo, J. A., Schongart, J., Jimene, and J. C. 2022. The new historical flood of 2021 in the Amazon River compared to major floods of the 21st century: Atmospheric features in the context of the intensification of floods. Weather and Clim. Extr. 35:100406. doi: 10.1016/j.wace.2021.100406.
8. Jones, E. F., Griffin, N., Kelso, J. E., Carling, G. T., Baker, M. A., and Aanderud, Z. T. 2020. Stream microbial community structured by trace elements, headwater dispersal, and large reservoirs in sub-alpine and urban ecosystems. Front. in Microbiol. 11:491425. doi: 10.3389/fmicb.2020.491425.
9. Kamimura, N. and others. 2017. Bacterial catabolism of lignin-derived aromatics: New findings in a recent decade: Update on bacterial lignin catabolism. Env. Microbiol. Rep. 9(6):679-705.
10. Kolbe, T., and others. 2019. Stratification of reactivity determines nitrate removal in groundwater. Proc. Natl. Acad. Sci. U.S.A. 116: 2494–2499. doi: 10.1073/pnas.1816892116.
11. Niño-García, J. P., Ruiz-González, C., and del Giorgio, P. A. 2016. Interactions between hydrology and water chemistry shape bacterioplankton biogeography across boreal freshwater networks. ISME J. 10: 1755–1766. doi: 10.1038/ismej.2015.226.
12. Oldham, C. E., Farrow, D. E., and Peiffer, S. 2013. A generalized Damköhler number for classifying material processing in hydrological systems. Hydrol. Earth Syst. Sci. 17: 1133–1148. doi: 10.5194/hess-17-1133-2013.
13. Santos, C. D. and others. 2017. Metagenome Sequencing of Prokaryotic Microbiota Collected from Rivers in the Upper Amazon Basin. Microbiol. Res. Ann. 5(2). doi: 10.1128/genomeA.01450-16.
14. Santos, C. D., Sarmento, H., de Miranda, F. P., Henrique-Silva, F., and Logares, R. 2020. Uncovering the genomic potential of the Amazon River microbiome to degrade rainforest organic matter. Microbiome. 8(1). doi: 10.1186/s40168-020-00930-w.

15. Santos, C. D., Toyama, D., de Oliveira, T. C. S., de Miranda, F. P., and Henrique-Silva, F. 2019. Flood season microbiota from the Amazon Basin lakes: Analysis with metagenome sequencing. Microbiol. Res. Ann. 8(17). doi: 10.1128/MRA.00229-19.
16. Satinsky, B. M. and others. 2014. The Amazon continuum dataset: quantitative metagenomic and metatranscriptomic inventories of the Amazon River plume, June 2010. Microbiome. 2. doi: 10.1186/2049-2618-2-17.
17. Segura, A., Bünz, P. V., D'Argenio, D. A., and Ornston, L. N. 1999. Genetic analysis of a chromosomal region containing vanA and vanB, genes required for conversion of either ferulate or vanillate to protocatechuate in *Acinetobacter*. J. Bacteriol. 181: 3494–3504. doi: 10.1128/JB.181.11.3494-3504.1999.
18. Toyama, D. and others. 2016. Metagenomics Analysis of Microorganisms in Freshwater Lakes of the Amazon Basin. Microbiol. Res. Ann. 4(6). doi: 10.1128/genomeA.01440-16.
19. Vetting, M. W., D'Argenio, D. A., Ornston, L. N., and Ohlendorf, D. H. 2000. Structure of *Acinetobacter* strain ADP1 protocatechuate 3, 4-dioxygenase at 2.2 A resolution: implications for the mechanism of an intradiol dioxygenase. Biochemistry. 39(27): 7943–7955. doi: 10.1021/bi000151e.
20. Zarnetske, J. P., Haggerty, R., Wondzell, S. M., and Baker, M. A. 2011. Dynamics of nitrate production and removal as a function of residence time in the hyporheic zone. J. Geophys. Res. Biogeosci. 116:G01025. doi: 10.1029/2010JG001356.
